# Derivation and Validation of Novel Phenotypes of Multiple Organ Dysfunction Syndrome in Critically Ill Children

**DOI:** 10.1001/jamanetworkopen.2020.9271

**Published:** 2020-08-11

**Authors:** L. Nelson Sanchez-Pinto, Emily K. Stroup, Tricia Pendergrast, Neethi Pinto, Yuan Luo

**Affiliations:** 1 Critical Care, Department of Pediatrics, Feinberg School of Medicine, Northwestern University, Chicago, Illinois; 2 Health and Biomedical Informatics, Department of Preventive Medicine, Feinberg School of Medicine, Northwestern University, Chicago, Illinois; 3 Division of Critical Care Medicine, Ann and Robert H. Lurie Children’s Hospital of Chicago, Chicago, Illinois; 4 Driskill Graduate Program, Feinberg School of Medicine, Northwestern University, Chicago, Illinois; 5 Section of Critical Care, Department of Pediatrics, The University of Chicago, Chicago, Illinois

## Abstract

**Importance:**

Multiple organ dysfunction syndrome (MODS) is a dynamic and heterogeneous process associated with high morbidity and mortality in critically ill children.

**Objective:**

To determine whether data-driven phenotypes of MODS based on the trajectories of 6 organ dysfunctions have prognostic and therapeutic relevance in critically ill children.

**Design, Setting, and Participants:**

This cohort study included 20 827 pediatric intensive care encounters among 14 285 children admitted to 2 large academic pediatric intensive care units (PICUs) between January 2010 and August 2016. Patients were excluded if they were older than 21 years or had undergone cardiac surgery. The 6 subscores of the pediatric Sequential Organ Failure Assessment (pSOFA) score were calculated for the first 3 days, including the subscores for respiratory, cardiovascular, coagulation, hepatic, neurologic, and renal dysfunctions. MODS was defined as a pSOFA subscore of at least 2 in at least 2 organs. Encounters were split in a 80:20 ratio for derivation and validation, respectively. The trajectories of the 6 subscores were used to derive a set of data-driven phenotypes of MODS using subgraph-augmented nonnegative matrix factorization in the derivation set. Data analysis was conducted from March to October 2019.

**Exposures:**

The primary exposure was phenotype membership. In the subset of patients with vasoactive-dependent shock, the interaction between hydrocortisone and phenotype membership and its association with outcomes were examined in a matched cohort.

**Main Outcomes and Measures:**

The primary outcome was in-hospital mortality. Secondary outcomes included persistent MODS on day 7, and vasoactive-free, ventilator-free, and hospital-free days. Regression analysis was used to adjust for age, severity of illness, immunocompromised status, and study site.

**Results:**

There were 14 285 patients with 20 827 encounters (median [interquartile range] age 5.2 years [1.5-12.7] years; 11 409 [54.8%; 95% CI, 54.1%-55.5%] male patients). Of these, 5297 encounters (25.4%; 95% CI, 24.8%-26.0%) were with patients who had MODS, of which 5054 (95.4%) met the subgraph count threshold and were included in the analysis. Subgraph augmented nonnegative matrix factorization uncovered 4 data-driven phenotypes of MODS, characterized by a combination of neurologic, respiratory, coagulation, and cardiovascular dysfunction, as follows: phenotype 1, severe, persistent encephalopathy (1019 patients [19.2%]); phenotype 2, moderate, resolving hypoxemia (1828 patients [34.5%]); phenotype 3, severe, persistent hypoxemia and shock (1012 patients [19.1%]); and phenotype 4, moderate, persistent thrombocytopenia and shock (1195 patients [22.6%]). These phenotypes were reproducible in a validation set of encounters, had distinct clinical characteristics, and were independently associated with outcomes. For example, using phenotype 2 as reference, the adjusted hazard ratios (aHRs) for death by 28 days were as follows: phenotype 1, aHR of 3.0 (IQR, 2.1-4.3); phenotype 3, aHR of 2.8 (IQR, 2.0-4.1); and phenotype 4, aHR of 1.8 (IQR, 1.2-2.6). Interaction analysis in a matched cohort of patients with vasoactive-dependent shock revealed that hydrocortisone had differential treatment association with vasoactive-free days across phenotypes. For example, patients in phenotype 3 who received hydrocortisone had more vasoactive-free days than those who did not (23 days vs 18 days; *P *for interaction < .001), whereas patients in other phenotypes who received hydrocortisone either had no difference or had less vasoactive-free days.

**Conclusions and Relevance:**

In this study, data-driven phenotyping in critically ill children with MODS uncovered 4 distinct and reproducible phenotypes with prognostic relevance and possible therapeutic relevance. Further validation and characterization of these phenotypes is warranted.

## Key Points

**Question:**

Does data-driven phenotyping based on the trajectories of organ dysfunction in the acute phase of critical illness among children with multiple organ dysfunction syndrome uncover phenotypes with prognostic and therapeutic relevance?

**Findings:**

In this 2-center cohort study of 20 827 pediatric intensive care encounters, a data-driven approach to phenotyping patients with multiple organ dysfunction syndrome using the trajectories of 6 organ dysfunctions uncovered 4 reproducible and distinct phenotypes with prognostic and potential therapeutic relevance.

**Meaning:**

In this study, data-driven phenotyping based on the type, severity, and trajectory of 6 organ dysfunctions showed promising results in critically ill children with multiple organ dysfunction syndrome.

## Teaser

This cohort study determines whether data-driven phenotypes of MODS based on the trajectory of 6 organ dysfunctions have prognostic and therapeutic relevance in critically ill children.

## Introduction

The development of multiple organ dysfunction syndrome (MODS) is a common final pathway for death among critically ill children.^1^ Even among survivors, children with more severe organ dysfunction are at higher risk of developing long-term morbidity after critical illness.^2^ Approximately one-third of children admitted to a pediatric intensive care unit (PICU) have MODS on presentation or develop it during their illnesses, so moderating the burden of MODS could significantly affect the outcomes of critically ill children.^1,3,4,5^

MODS can develop after many types of injury, but sepsis, trauma, and major surgery are the most common etiologies in the PICU.^1,6,7^ The most frequently described shared mechanism of MODS pathophysiology is the development of dysregulated inflammation; however, decades of drug trials targeting mediators of inflammation have failed to show effectiveness in the reduction of the MODS burden associated with sepsis and trauma.^7,8,9,10,11^ One of the most likely explanations for this is that MODS is a complex, dynamic, and heterogeneous process with many different phenotypes, and no single strategy will be effective in all phenotypes of MODS.

In recent years, considerable emphasis has been placed on characterizing the phenotypes of heterogenous syndromes in critically ill patients.^12,13,14,15,16,17^ These phenotypes may have prognostic and therapeutic implications and form the basis for precision medicine in the critical care setting.^18^ The derivation of these different phenotypes has included data-driven approaches using gene expression data^12,17^ and clinical data^14,15,16^ as well as expert-based approaches using biomarkers and clinical data.^13^ In general, these approaches have used a single time (eg, initial presentation) to define the different phenotypes and have not investigated the dynamic patterns of illness, despite evidence that this may be important in phenotyping.^19,20,21,22^ Furthermore, a single snapshot approach does not account for the fact that patients may present to a critical care setting at different points in their illness and that organ dysfunctions tend to peak between days 1 and 3 of admission.^23^

In this study we aimed to derive, validate, and characterize novel phenotypes of MODS in critically ill children using a data-driven approach based on the type, severity, and trajectory of organ dysfunctions in the acute phase of critical illness. Furthermore, we aimed to determine whether these phenotypes had prognostic and therapeutic relevance.

## Methods

### Study Design and Population

This was a retrospective cohort study of critically ill children admitted to 2 large, academic PICUs in Chicago, Illinois, between January 1, 2010, and August 31, 2016. Patients were excluded if they were older than 21 years or were recovering from cardiac surgery. Data were extracted from the 2 institutions’ data warehouses using structured queries and underwent quality checks for conformity, completeness, and plausibility. Data analysis was conducted from March to October 2019. The institutional review boards at the Ann and Robert H. Lurie Children’s Hospital of Chicago and The University of Chicago approved this study with a waiver of informed consent because of the retrospective nature of the analysis with minimal risk to patients. The reporting of this cohort study was performed using the Strengthening the Reporting of Observational Studies in Epidemiology (STROBE) reporting guideline.^37^

Organ dysfunction severity was measured using the subscores of the pediatric Sequential Organ Failure Assessment (pSOFA) score, which measures dysfunction for the respiratory, cardiovascular, coagulation, hepatic, neurologic, and renal systems on a scale from 0 to 4 for each system.^24^ Patients had the 6 pSOFA subscores calculated for each 24-hour period between PICU admission and day 3, which was considered the acute phase of critical illness.^23^ Individual pSOFA subscores were carried forward for as long as 24 hours if they were not remeasured; otherwise, missing variables were assumed to be normal and the corresponding subscore was assigned a 0.

Patients with MODS were defined as those with a pSOFA subscore of at least 2 in at least 2 organ systems within the first 3 days of admission. Severity of illness on admission was determined using the Pediatric Risk of Mortality (PRISM) III score using variables from the first 24 hours.^25^ Chronic comorbidities were based on the classification system developed by Feudtner et al.^26^ Patients with immunocompromised status were defined as those with an oncologic disease or transplant recipients. Patients who received antibiotics and microbiological cultures during the first 3 days were considered to have a confirmed or suspected infection. Each patient encounter was treated independently, but only the first PICU admission in a given hospitalization was included in the analysis.

### Primary and Secondary Outcomes

The primary outcome was in-hospital mortality. The secondary outcomes included the presence of persistent MODS on day 7 after PICU admission (which included patients who died in the first week), and vasoactive-free, ventilator-free, and hospital-free days at 28 days after PICU admission.

### Data-Driven Phenotyping

**Figure 1.**
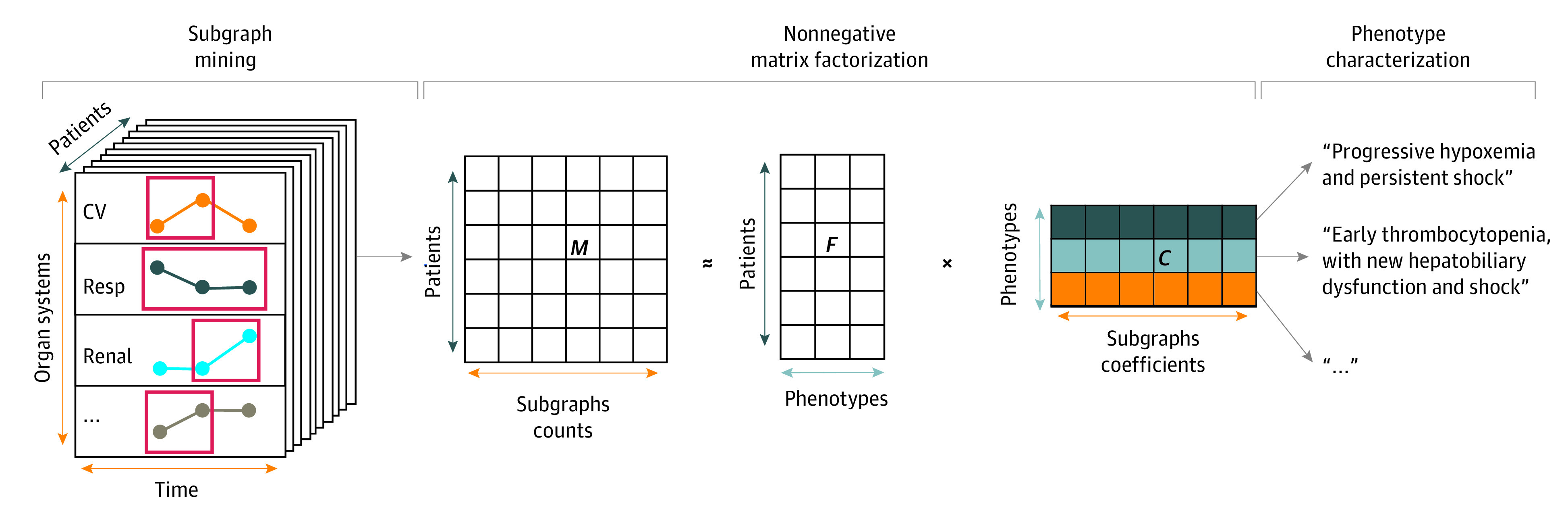
Data-Driven Phenotyping Using Subgraph Augmented Nonnegative Matrix Factorization Subgraph mining extracts representative subgraphs from the trajectories of the 6 pediatric Sequential Organ Failure Assessment subscores in patients with MODS, which results in a matrix, *M*, of patient-subgraph counts. Nonnegative matrix factorization is used to derive a matrix, *F*, of hidden features (in this case the phenotypes), and a matrix of mixture coefficient *C* with the coefficients that compose the subgraph-based phenotypes. The factorization is done by iteratively updating *F* and *C* using the sparse nonnegative matrix factorization with sparseness in the left factor algorithm to gradually reduce the error between *M* and *F* × *C*. Once the nonnegative matrix factorization is completed, the final phenotypes can be characterized given the highly interpretable nature of the algorithm. Of note, the size of the matrices, number of groups, and the phenotype characterization examples here are used for illustration purposes only. CV indicates cardiovascular; resp, respiratory.

We used subgraph-augmented nonnegative matrix factorization to group patients with MODS into phenotypes based on the type, severity, and trajectory of each of the 6 pSOFA subscores in the first 3 days of PICU stay (Figure 1; eAppendix in the Supplement).^21^ Briefly, subgraph mining was used to extract the subgraphs representing the trajectory of individual organ dysfunctions.^27^ We then split the cohort of 5054 encounters with MODS and adequate subgraph counts into a derivation set (4044 encounters [80.0%]) and a validation set (1010 encounters [20.0%]). Nonnegative matrix factorization (NMF) was implemented on the patient-subgraph count matrix in the derivation set, which resulted in a patient-phenotype distribution matrix and a phenotype-subgraph mixture coefficient matrix.^21,28^ Patients in the derivation and validation set were then assigned phenotype membership based on the highest probability group in the mixture coefficient matrix. Patients assigned to each phenotype were then compared across the derivation and validation sets.

As a sensitivity analysis to determine external validity of the phenotypes, the same procedures were conducted using patients with MODS in the second hospital (PICU B) as the external validation set. Additional details can be found in the eAppendix in the Supplement.

### Phenotype Characteristics and Clinical Relevance Evaluation

#### Clinical Characteristics of the MODS Phenotypes

The clinical characteristics of patients in each of the MODS phenotypes were compared across phenotypes. These included demographic characteristics, severity of illness, organ support, comorbidities, microbiologic and laboratory test results by day 3, and associated outcomes. The laboratory tests and summary measures included were based on those frequently used in prior pediatric organ dysfunction and severity of illness scores.^3,25,29,30^

#### Prognostic Relevance of the MODS Phenotypes

The prognostic relevance of the MODS phenotypes was tested by comparing the association of each phenotype with the outcomes of interest after adjusting for common confounders of PICU-related morbidity and mortality, including age, immunocompromised state, severity of illness on admission using the PRISM-III score, and study site.^4,31^ The phenotype with the lowest overall in-hospital mortality was used as reference. Additionally, the association of noncharacteristic organ dysfunctions and in-hospital mortality was analyzed in each of the phenotypes. Noncharacteristic organ dysfunctions were defined as those organ systems that were not used to characterize a specific phenotype and had a pSOFA subscore of at least 2 by day 3 of PICU stay.

#### Therapeutic Relevance of the MODS Phenotypes in Patients With Vasoactive-Dependent Shock

There are few organ dysfunction–specific treatments routinely used in patients with MODS. One exception is the use of intravenous (IV) hydrocortisone in patients with vasoactive-dependent shock. Hydrocortisone may contribute to hemodynamic stability through pleiotropic effects; however, mixed results in large randomized clinical trials among adults with septic shock have led to variable use among practitioners.^32,33,34,35,36^ Given that shock is a common feature of MODS, we tested the hypothesis that the heterogeneity of treatment effect of IV hydrocortisone in patients with vasoactive-dependent shock is in part explained by their phenotype. Vasoactive-dependent shock was defined as the use of dopamine greater than 5 µg/kg/min or any dose of epinephrine or norepinephrine infusions in patients with MODS. To balance the confounders across treatment groups, we first performed propensity score matching of patients based on the propensity to receive IV hydrocortisone, using age, immunocompromised state, PRISM-III score, study site, and phenotype membership as covariates. We then performed an interaction effect analysis for in-hospital mortality and vasoactive-free days, using phenotype membership, treatment group (ie, receiving ≥2 mg/kg/d of IV hydrocortisone or not), and their interaction as covariates to determine whether there was a differential treatment effect associated with phenotype membership. Additional details can be found in the eAppendix in the Supplement.

### Statistical Analysis

Data were analyzed using R version 3.6.1 (R Project for Statistical Computing). Categorical variables were compared using the χ^2^ test and continuous variables using the Kruskal-Wallis test. Regression analysis was used to adjust for confounders when comparing outcomes. Logistic regression was used to compare binary outcomes, and Poisson regression was used to compare count outcomes. Survival analysis to 28 days was performed using Kaplan-Meier curves and adjusted Cox regression analysis. Statistical significance was set at *P* < .05, and all tests were 2-tailed.

## Results

### Study Population

There were 20 827 patient encounters among 14 285 unique patients in the 2 PICUs during the study period. The median (interquartile range [IQR]) age was 5.2 (1.5-12.7) years, and 11 409 (54.8%; 95% CI, 54.1%-55.5%) were male patients. A total of 5297 patients (25.4%; 95% CI, 24.8%-26.0%) met MODS criteria, and these patients had an associated in-hospital mortality of 449 (8.5%; 95% CI, 7.7%-9.3%). The clinical characteristics and outcomes of patients with and without MODS are presented in Table 1.

**Table 1.** Demographic and Clinical Characteristics of Patients With and Without MODS

Clinical variables	Patients, No. (%)		*P* value
	With MODS (n = 5297)	Without MODS (n = 15 530)	
Age, median (IQR), y	4.7 (1.0-12.6)	5.4 (1.6-12.7)	<.001
Male patients	2898 (54.7)	8511 (54.8)	.90
Race/ethnicity			
Non-Hispanic			<.001
White	1700 (32.1)	4966 (32)	
Black	1726 (32.6)	5515 (35.5)	
Hispanic	1342 (25.3)	3650 (23.5)	
Other	529 (10)	1399 (9)	
PRISM-III score on admission, median (IQR)	7 (4-13)	0 (0-4)	<.001
Vasoactives by day 3	1222 (23.1)	84 (0.5)	<.001
Mechanical ventilation by day 3	3883 (73.3)	3108 (20)	<.001
Immunocompromised state	922 (17.4)	1422 (9.2)	<.001
Multiple chronic comorbidities	3153 (59.5)	5806 (37.3)	<.001
Postoperative	1286 (24.3)	3679 (23.7)	.40
Trauma	200 (3.8)	827 (5.3)	<.001
Infection by day 3	3145 (59.4)	4665 (30)	<.001
Maximum pSOFA score by day 3, median (IQR)	7 (6-9)	2 (1-3)	<.001
Maximum PELOD-2 score by day 3, median (IQR)	7 (5-10)	2 (2-4)	<.001
MODS on day 7	1418 (26.8)	124 (0.8)	<.001
Length of stay, median (IQR), d	9.1 (4.8-17.9)	3.2 (1.8-5.9)	<.001
In-hospital mortality	449 (8.5)	73 (0.5)	<.001

Abbreviations: IQR, interquartile range; MODS, multiple organ dysfunction syndrome; PRISM-III, Pediatric Risk of Mortality III; pSOFA, pediatric Sequential Organ Failure Assessment; PELOD-2, Pediatric Logistic Organ Dysfunction, version 2.

### Data-Driven Phenotyping

After performing subgraph mining of the pSOFA subscore trajectories during the first 3 days among patients with MODS and eliminating redundant, rare, and nonrepresentative subgraphs based on the criteria described in the eAppendix in the Supplement, there were 145 representative subgraphs uncovered. A total of 243 patient encounters (4.6%) with MODS (with an in-hospital mortality of 3 [1.2%]) had none of these representative subgraphs and were therefore excluded from the remainder of the analysis, resulting in 5054 encounters (95.4%) with patients with MODS in the final cohort. Of the 145 subgraphs representing pSOFA subscore trajectories in these 5054 encounters, 31 (21.4%) represented worsening organ dysfunction, 24 (16.6%) represented moderate-to-severe organ dysfunction with no change, and 90 (62.1%) represented improvement in organ dysfunction.

Sparse NMF was performed in the derivation set (4044 encounters [80.0%]) using the counts of the 145 subgraphs as sole input. This uncovered 4 distinct phenotypes based on the changes in pSOFA subscore in the first 3 days. Phenotype membership was then assigned to patients in the entire cohort based on the highest probability group membership according to the mixture coefficient matrix. Patients in each phenotype were then compared across the derivation and validation sets. Within each of the 4 phenotypes, patients had similar distributions of pSOFA subscores on day 1 to day 3 (eTable 1 in the Supplement), laboratory test characteristics by day 3 (eTable 2 in the Supplement), and outcomes (eTable 3 in the Supplement).

In the sensitivity analysis using 3013 patient encounters (59.6%) in PICU A as derivation and 2041 patient encounters (40.4%) in PICU B as validation, again sparse NMF uncovered 4 phenotypes, which were consistent with those in the original analysis. The distributions of pSOFA subscores, laboratory test results, and outcomes were similar within phenotypes across the derivation patient encounters in PICU A and the external validation patient encounters in PICU B (eTable 4 in the Supplement). Patients within each phenotype from the original NMF were merged and used for the remainder of the analyses.

### Characterization of the MODS Phenotypes

**Figure 2.**
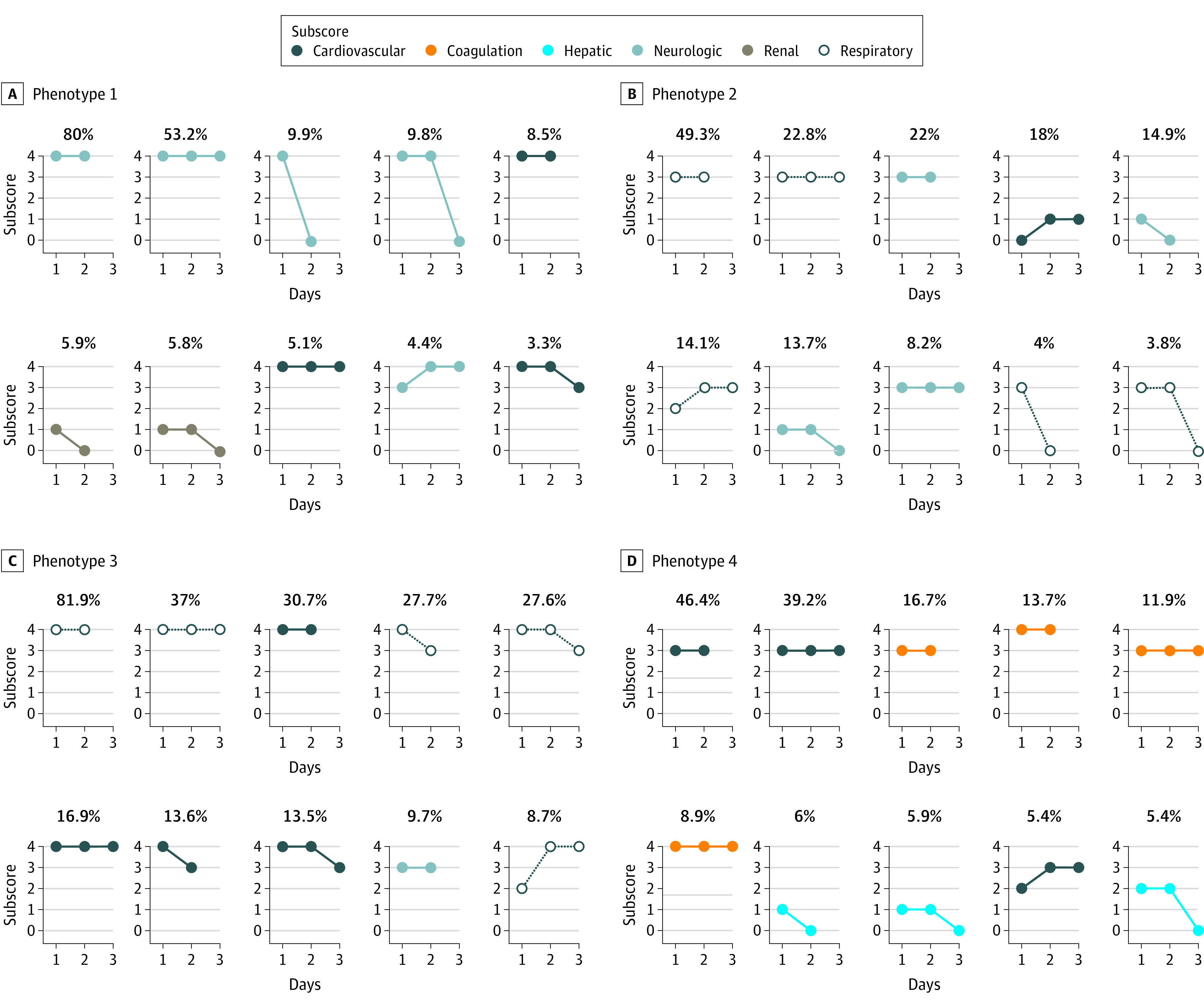
Common Subgraphs in Each of the 4 Data-Driven MODS Phenotypes The most common subgraphs in each phenotype and the proportion of patients who had each of them.

The most representative subgraphs for each phenotype were chosen from the mixture coefficient matrix of the NMF model, such that the selected subgraphs accounted for at least 75% of the phenotype group coefficients. The 10 most representative subgraphs for each of the 4 MODS phenotypes and the relative weight of each organ dysfunction subscore in the first 3 days, stratified by phenotype, appear in Figure 2 and eFigure 1 in the Supplement. These representative subgraphs were used to characterize the phenotypes based on the most distinctive characteristics, using the organ dysfunction type, severity, and trajectory in the first 3 days of PICU stay. Based on this, the 4 MODS phenotypes were characterized as follows: phenotype 1, severe, persistent encephalopathy (1019 patients [19.2%]); phenotype 2, moderate, resolving hypoxemia (1828 patients [34.5%]); phenotype 3: severe, persistent hypoxemia and shock (1012 patients [19.1%]); and phenotype 4, moderate, persistent thrombocytopenia and shock (1195 patients [22.6%])

The clinical characteristics and associated outcomes of patients in each of the 4 MODS phenotypes are presented in Table 2. When compared across age groups, the distribution and outcomes associated with the 4 MODS phenotypes remained consistent (eTable 5 in the Supplement).

**Table 2.** Demographic and Clinical Characteristics and Outcomes of the 4 MODS Phenotypes

Clinical variable	No. (%)				*P* value
	Phenotype 1, severe, persistent encephalopathy (n = 1019)	Phenotype 2, moderate, resolving hypoxemia (n = 1828)	Phenotype 3, severe, persistent hypoxemia and shock (n = 1012)	Phenotype 4, moderate, persistent thrombocytopenia and shock (n = 1195)	
Age, median (IQR), y	4.3 (1.0-12.0)	3.9 (0.9-11.3)	3.5 (0.8-11.3)	8.1 (1.8-15.2)	<.001
Male patients	555 (54.5)	989 (54.1)	557 (55.0)	671 (56.2)	.73
Race/ethnicity					
Non-Hispanic					<.001
White	311 (30.5)	557 (30.5)	319 (31.5)	447 (37.4)	
Black	410 (40.2)	636 (34.8)	318 (31.4)	285 (23.8)	
Hispanic	197 (19.3)	453 (24.8)	265 (26.2)	353 (29.5)	
Other	101 (9.9)	182 (10)	110 (10.9)	110 (9.2)	
PRISM-III score on admission, median (IQR)	8 (5-15)	6 (2-10)	10 (5-19)	9 (5-13)	<.001
Vasoactives by day 3	179 (17.6)	107 (6)	392 (38.7)	542 (45.3)	<.001
Mechanical ventilation by day 3	926 (91)	1409 (77.1)	880 (87)	553 (46.3)	<.001
Immunocompromised state	105 (10.3)	240 (13.1)	154 (15.2)	382 (32)	<.001
Multiple chronic comorbidities	607 (59.6)	1043 (57.1)	608 (60)	760 (63.4)	.004
Postoperative	223 (21.9)	527 (28.8)	164 (16.2)	307 (25.7)	<.001
Trauma	72 (7.1)	55 (3)	41 (4.1)	30 (2.5)	<.001
Infection by day 3	595 (58.4)	953 (52.1)	724 (71.5)	755 (63.2)	<.001
Primary site					
Bloodstream	59 (5.8)	117 (6.4)	119 (11.2)	193 (16.2)	<.001
Respiratory	334 (32.8)	510 (27.9)	385 (38)	193 (16.2)	<.001
Urinary	66 (6.5)	96 (5.3)	62 (6.1)	81 (6.8)	.32
Other sites	36 (3.5)	60 (3.3)	35 (3.5)	45 (3.8)	.92
Culture negative	194 (19)	308 (16.8)	252 (24.9)	328 (27.4)	<.001
Microorganism					
Bacteria					
Gram positive	133 (13.1)	184 (10.1)	159 (15.7)	127 (10.6)	<.001
Gram negative	165 (16.2)	224 (12.3)	178 (17.6)	137 (11.5)	<.001
Other bacteria	32 (3.1)	50 (2.7)	22 (2.2)	39 (3.3)	.42
Fungi	12 (1.2)	17 (0.9)	18 (1.8)	21 (1.8)	.14
Viruses	170 (16.7)	333 (18.2)	230 (22.7)	184 (15.4)	<.001
Laboratory results by day 3, median (IQR)					
Minimum white blood cell count, /μL	7900 (5200-11 000)	8300 (5600-11 600)	7000 (3800-10 900)	5900 (2500-9400)	<.001
Minimum lymphocyte count, /μL	1300 (700-2200)	1300 (700-2300)	1100 (500-2000)	800 (300-1500)	<.001
Maximum blood urea nitrogen, mg/dL	12 (8-18)	12 (8-18)	14 (9-23)	15 (10-28)	<.001
Maximum creatinine, mg/dL	0.4 (0.3-0.6)	0.4 (0.2-0.6)	0.4 (0.3-0.8)	0.5 (0.3-0.9)	<.001
Minimum albumin, g/dL	3.1 (2.5-3.6)	3.1 (2.6-3.7)	2.7 (2.2-3.3)	2.8 (2.3-3.4)	<.001
Maximum aminotransferase, U/L					
Aspartate	53 (31-147)	49 (30-111)	61 (34-201)	62 (32-141)	<.001
Alanine	28 (18-17)	27 (17-71)	36 (19-122)	37 (18-97)	<.001
Maximum total bilirubin, mg/dL	0.5 (0.3-1.2)	0.5 (0.2-2.2)	0.6 (0.3-1.5)	1.2 (0.5-3.2)	<.001
Minimum hemoglobin, g/dL	9 (7.4-11)	9.6 (7.8-11.3)	8.8 (7.2-10.5)	8.3 (7-10)	<.001
Minimum platelet count, /μL	185 000 (104 000-268 000)	188 000 (94 000-270 000)	156 000 (68 000-249 000)	82 000 (27 000-179 000)	<.001
Maximum international normalized ratio	1.4 (1.2-1.7)	1.3 (1.2-1.6)	1.5 (1.2-2.1)	1.4 (1.2-1.7)	<.001
Maximum partial thromboplastin time, s	35 (30-45)	35 (30-44)	40 (32-59)	39 (31-54)	<.001
Maximum lactate, mg/dL	18.9 (10.8-35.1)	14.4 (9.9-26.1)	22.5 (12.6-52.2)	18.0 (11.7-34.2)	<.001
Outcomes					
Vasoactive-free days at 28 d, median (IQR)	28 (27-28)	28 (28-28)	28 (24-28)	27 (26-28)	<.001
Ventilator-free days at 28 d, median (IQR)	23 (13-26)	26 (21-27)	20 (6-26)	27 (22-28)	<.001
Hospital-free days at 28 d, median (IQR)	16 (1-23)	20 (12-24)	12 (1-21)	17 (4-22)	<.001
Persistent MODS on day 7	320 (31.4)	319 (17.5)	448 (44.3)	307 (25.7)	<.001
In-hospital mortality	116 (11.3)	60 (3.3)	176 (17.4)	94 (7.9)	<.001

Abbreviations: IQR, interquartile range; MODS, multiple organ dysfunction syndrome; PRISM-III, Pediatric Risk of Mortality III.

SI conversion factors: To convert alanine aminotransferase and aspartate aminotransferase to microkatals per liter, multiply by 0.0167; albumin and hemoglobin to grams per liter, multiply by 10; blood urea nitrogen to millimoles per liter, multiply by 0.357; creatinine to micromoles per liter, multiply by 88.4; lymphocyte, platelet, and white blood cell count to ×10^9^ per microliter, multiply by 0.001; and total bilirubin to millimoles per liter, multiply by 17.104.

### Prognostic Relevance of the MODS Phenotypes

The outcomes associated with each of the 4 phenotypes after adjusting for confounders including age, immunocompromised state, PRISM-III score, and study site are presented in eTable 6 in the Supplement. After adjusting for the same confounders in Cox regression analysis and using phenotype 2, which had the lowest mortality as reference, the adjusted hazard ratios (aHRs) for death by 28 days were as follows: phenotype 1, aHR of 3.0 (IQR, 2.1-4.3); phenotype 3, aHR of 2.8 (IQR, 2.0-4.1); and phenotype 4, aHR of 1.8 (IQR, 1.2-2.6). The Kaplan-Meier survival curves for the 4 phenotypes are presented in eFigure 2 in the Supplement. The multivariable logistic regression analyses of the association of noncharacteristic organ dysfunctions and in-hospital mortality showed significant differences between phenotypes and are presented in eTable 7 in the Supplement.

### Therapeutic Relevance of the MODS Phenotypes in Patients With Vasoactive-Dependent Shock

A total of 1220 of 5054 patient encounters (24.1%; 95% CI, 23.0%-25.3%) had vasoactive-dependent shock by day 3, with an associated in-hospital mortality of 299 patients (24.5% [95% CI, 22.1%-27%]). Of these, 342 (28.0%; 95% CI, 25.5%-30.6%) received IV hydrocortisone. Using propensity score matching, 330 treated patients were successfully matched 1:1 with 330 untreated patients using age, immunocompromised state, PRISM-III score, study site, and phenotype membership as covariates. The matched cohort demonstrated a balanced distribution of covariates, with absolute standardized mean difference of less than 0.1 (eFigure 3 in the Supplement). Interaction analysis in the matched cohort revealed that IV hydrocortisone had a differential treatment effect on vasoactive-free days across phenotypes, most notably with phenotype 3 having most benefit associated with treatment (23 vasoactive-free days in treated patients vs 18 vasoactive-free days in treated patients; *P* for interaction < .001). The interaction was not significant for in-hospital mortality (Table 3).

**Table 3.** Differences in Response to IV Hydrocortisone by Phenotype in a Propensity Score Matched Cohort of Patients With Vasoactive-Dependent Shock

Outcome	Phenotype 1 (n = 72)		Phenotype 2 (n = 71)		Phenotype 3 (n = 287)		Phenotype 4 (n = 230)		*P* value^a^
	Treated (n = 39)	Untreated (n = 33)	Treated (n = 36)	Untreated (n = 35)	Treated (n = 146)	Untreated (n = 141)	Treated (n = 109)	Untreated (n = 121)	
Vasoactive-free days, median (IQR)	15 (0-25)	17 (1-24)	25 (23-26)	24 (21-27)	23 (1-26)	18 (0-26)	26 (23-27)	26 (24-27)	<.001
In-hospital mortality, No.(%)	20 (51.3)	14 (42.4)	4 (11.1)	4 (11.4)	50 (34.2)	65 (46.1)	14 (12.8)	9 (7)	.11

Abbreviations: IQR, interquartile range; IV, intravenous.

^a^ *P* value for interaction between IV hydrocortisone and phenotype membership using phenotype 2 as reference group (ie, 3 interaction terms) and analysis of deviance to test global significance.

## Discussion

We derived and validated 4 novel, data-driven phenotypes of MODS in critically ill children based on organ dysfunction trajectories. These phenotypes have distinct clinical characteristics, are independently associated with outcomes, and have different sets of organ dysfunction–based risk factors for death. In a subset of patients with vasoactive-dependent shock who were matched based on the propensity to receive IV hydrocortisone, treatment was associated with a difference in vasoactive-free days across phenotypes, suggesting that these phenotypes may be associated with differences in response to therapy.

Our findings share similarities with previously described data-driven phenotypes of sepsis and sepsis-induced MODS, particularly phenotypes 3 and 4, which were the MODS phenotypes most frequently associated with infections in our cohort. Phenotype 3 shares similarities with the “shock with hypoxemia and altered mental status” phenotype described by Knox et al^15^ and the γ phenotype described by Seymour et al^14^ in adult patients, especially in terms of hypoxemia, vasoactive-dependence, low albumin levels, and high mortality. Phenotype 4 shares similarities with the hepatic disease phenotype described by Knox et al^15^ and the δ phenotype described by Seymour et al,^14^ particularly in terms of the thrombocytopenia, hepatobiliary dysfunction, and shock as well as the relatively preserved pulmonary function. To our knowledge, our study is the first to use a data-driven approach to derive MODS phenotypes in critically ill children. Further biomarker and molecular endotyping analyses will be needed to compare our phenotypes with other previously described clinical phenotypes and endotypes in pediatric and adult patients.^12,13,17^

The association of IV hydrocortisone administration with differential treatment effect on vasoactive-free days in patients with vasoactive-dependent shock, particularly in phenotype 3, could partially be explained by the enrichment with patients suffering severe hypoxemia, severe shock, and infections. Prior randomized clinical trials and secondary analyses of trial data have found that the use of hydrocortisone was associated with improved outcomes in patients with sepsis-associated acute respiratory distress syndrome.^38,39^ However, our results should be interpreted with caution because they are part of an interaction effect analysis on a propensity score matched cohort. The standard approach is to perform an interaction effects analysis across subgroups with a treatment assignment that has been randomized to ensure a better balance of all possible confounders.^16^ Given the selection bias inherent in observational data, we used propensity score matching to balance observed covariates, but it is likely that we did not account for all possible confounders. Therefore, these results require further validation and are only hypothesis generating at this point.

Our data-driven phenotyping approach to MODS has several important implications. We describe a broad stratification of patients that can be readily performed using widely available electronic health record data and that appears to have prognostic relevance and, potentially, therapeutic value. It is likely that many of the patients within each phenotype share many of the same underlying pathophysiological disturbances and dysregulated host responses, as evidenced by the shared clinical characteristics and laboratory test results. Understanding the common features of these underlying pathophysiologies could help determine which interventions may be most beneficial to individual patients, regardless of the etiology of MODS and beyond the traditional boundaries of syndromes like sepsis or acute respiratory distress syndrome. For example, cardiovascular dysfunction is associated with the most detrimental outcomes in patients with severe encephalopathy in phenotype 1, suggesting that targeted blood pressure management and close cerebral perfusion monitoring, as recommended for patients with traumatic brain injury, may be warranted in patients with this phenotype even if most do not have traumatic brain injury.^40^ Similarly, renal dysfunction seems to most negatively affect patients in phenotype 3 who suffer severe hypoxemia and shock, indicating that careful fluid management and nephrotoxin avoidance might be most beneficial in this group of patients. In addition, further endotyping and molecular characterization of these broad phenotypes may uncover relevant MODS subphenotypes that may benefit from novel targeted interventions, including immunomodulatory strategies.^41,42,43^ Furthermore, the molecular characterization of these phenotypes may help in the task of assigning phenotype membership shortly after patients are admitted, which would make this phenotyping schema more clinically actionable in the critical care setting. In short, data-driven phenotyping may help develop precision medicine strategies that can reduce the mortality and morbidity associated with MODS, but further investigation into the value of this phenotyping approach in research and clinical care is warranted.^18^

### Limitations

Our findings are subject to several limitations. First, the data for this study were collected retrospectively from 2 academic PICUs in the same city, limiting the generalizability of our findings. Second, our data-driven phenotyping approach was intentionally sparse, using only organ dysfunction trajectories to define broad phenotypes and determine their prognostic and therapeutic relevance, but performing deeper phenotyping using other clinical features and biomarkers may result in phenotypes with potentially higher resolution and greater clinical utility. Third, the therapeutic relevance of IV hydrocortisone was studied using an interaction effect analysis on a propensity score matched cohort, and the results must be interpreted with caution, as previously discussed. Fourth, although we described the MODS phenotypes as distinct entities, it is likely that many patients share features of more than 1 phenotype, and future analyses to better understand the implications of these overlaps are warranted.

## Conclusions

In this study, we derived and validated 4 data-driven phenotypes of MODS in critically ill children that have distinct clinical characteristics, are independently associated with outcomes, and may be therapeutically relevant in the subset of patients with vasoactive-dependent shock. Our data-driven phenotyping approach in MODS shows promising results, but further characterization of these broad phenotypes and validation in different settings is warranted.

## Notes

Supplement. **eAppendix. **Supplementary Methods **eFigure 1. **Alluvial Plots of Organ Dysfunction Burden by Day in the 4 MODS Phenotypes **eFigure 2. **Kaplan-Meier Survival Curves for the First 28 Days for the 4 MODS Phenotypes **eFigure 3. **Covariate Balance in the Propensity Score Matched Cohort of Patients With Vasoactive-Dependent Shock Who Received and Did Not Receive Intravenous Hydrocortisone **eTable 1. **Maximum pSOFA Subscores Achieved by Day in Patients Within Each of the 4 MODS Phenotypes in the Derivation and Validation Sets **eTable 2. **Laboratory Test Results by Day 3 of PICU Stay in the 4 MODS Phenotypes Across the Derivation and Validation Sets **eTable 3. **Outcomes Associated With the 4 MODS Phenotypes Across the Derivation and Validation Sets **eTable 4. **Laboratory Test Results and Outcomes Associated With the MODS Phenotypes When Using Patients in Hospital A as the Derivation Set and Patients in Hospital B as an External Validation Set **eTable 5. **Distribution and Outcomes Associated With the 4 MODS Phenotypes Across Age Groups **eTable 6. **Unadjusted and Adjusted Outcomes Associated With the 4 MODS Phenotypes **eTable 7. **Multivariable Association Between Noncharacteristic Organ Dysfunctions and In-Hospital Mortality **eReferences.**
