# Supplementary material for: Derivation and Validation of Novel Phenotypes of Multiple Organ Dysfunction Syndrome in Critically Ill Children
Source: JAMA Netw Open. 2020 Aug 11;3(8):e209271. doi: 10.1001/jamanetworkopen.2020.9271 (PMC7420303; doi:10.1001/jamanetworkopen.2020.9271)
Supplement: Supplement. — eAppendix. Supplementary Methods eFigure 1. Alluvial Plots of Organ Dysfunction Burden by Day in the 4 MODS Phenotypes eFigure 2. Kaplan-Meier Survival Curves for the First 28 Days for the 4 MODS Phenotypes eFigure 3. Covariate Balance in the Propensity Score Matched Cohort of Patients With Vasoactive-Dependent Shock Who Received and Did Not Receive Intravenous Hydrocortisone eTable 1. Maximum pSOFA Subscores Achieved by Day in Patients Within Each of the 4 MODS Phenotypes in the Derivation and Validation Sets eTable 2. Laboratory Test Results by Day 3 of PICU Stay in the 4 MODS Phenotypes Across the Derivation and Validation Sets eTable 3. Outcomes Associated With the 4 MODS Phenotypes Across the Derivation and Validation Sets eTable 4. Laboratory Test Results and Outcomes Associated With the MODS Phenotypes When Using Patients in Hospital A as the Derivation Set and Patients in Hospital B as an External Validation Set eTable 5. Distribution and Outcomes Associated With the 4 MODS Phenotypes Across Age Groups eTable 6. Unadjusted and Adjusted Outcomes Associated With the 4 MODS Phenotypes eTable 7. Multivariable Association Between Noncharacteristic Organ Dysfunctions and In-Hospital Mortality eReferences. [file jamanetwopen-3-e209271-s001.pdf]

Supplementary Online Content

Sanchez-Pinto LN, Stroup EK, Pendergrast T, Pinto N, Luo Y. Derivation and validation of novel phenotypes of multiple organ dysfunction syndrome in critically ill children. *JAMA Netw Open*. 2020;3(8):e209271. doi:10.1001/jamanetworkopen.2020.9271

eAppendix. Supplementary Methods
eFigure 1. Alluvial Plots of Organ Dysfunction Burden by Day in the 4 MODS Phenotypes
eFigure 2. Kaplan-Meier Survival Curves for the First 28 Days for the 4 MODS Phenotypes
eFigure 3. Covariate Balance in the Propensity Score Matched Cohort of Patients With Vasoactive-Dependent Shock Who Received and Did Not Receive Intravenous Hydrocortisone
eTable 1. Maximum pSOFA Subscores Achieved by Day in Patients Within Each of the 4 MODS Phenotypes in the Derivation and Validation Sets
eTable 2. Laboratory Test Results by Day 3 of PICU Stay in the 4 MODS Phenotypes Across the Derivation and Validation Sets
eTable 3. Outcomes Associated With the 4 MODS Phenotypes Across the Derivation and Validation Sets
eTable 4. Laboratory Test Results and Outcomes Associated With the MODS Phenotypes When Using Patients in Hospital A as the Derivation Set and Patients in Hospital B as an External Validation Set
eTable 5. Distribution and Outcomes Associated With the 4 MODS Phenotypes Across Age Groups
eTable 6. Unadjusted and Adjusted Outcomes Associated With the 4 MODS Phenotypes
eTable 7. Multivariable Association Between Noncharacteristic Organ Dysfunctions and In-Hospital Mortality
eReferences.

This supplementary material has been provided by the authors to give readers additional information about their work.

© 2020 Pinto-Sanchez LN et al. *JAMA Network Open.*

eAppendix. Supplementary Methods

Expanded data-driven phenotyping methods

Overview. Unsupervised learning algorithms are a subset of machine learning algorithms that are used to uncover naturally occurring patterns, groupings, or clusters in data, without targeting a specific outcome.[1] The most compelling use case of unsupervised learning in healthcare is in precision medicine, where the goal is to uncover subsets of patients who share similar characteristics and are, in theory, more likely respond to targeted therapies and share similar fates. We define data-driven phenotyping as the task of uncovering natural groups of patients based on their shared clinical characteristics using unsupervised learning algorithms. Non-negative matrix factorization (NMF) is an algorithm with inherent clustering properties that is designed to explain the observed data using a limited number of hidden features (or basis components), which when combined together using mixture coefficient matrices approximate the original data as accurately as possible.[2,3] Additionally, NMF-derived factors are simple, additive, and intuitive, which greatly increases their interpretability. In the biomedical context, NMF has been used to cluster cell lines, microarray data, tumor types, and critically ill patients.[4-7] Subgraph augmented NMF (SANMF) enhances NMF by incorporating subgraph mining and can be used to group temporal trends in clinical data.[7] Subgraph mining is a technique for pattern mining that in the biomedical context has also been used primarily in natural language processing applications.[8,9]

Given the dynamic nature of MODS, SANMF allowed us to perform data-driven phenotyping of patients by modeling the trajectories of organ dysfunctions based on the changes in the pSOFA subscores over the first 3 days of PICU stay (Figure 1). In our approach, subgraph mining was use to extract representative subgraphs from the trajectories of the six pSOFA subscores in patients with MODS, which resulted in a matrix M of patients-subgraphs counts. NMF was then used to derive a matrix F of hidden features, which corresponded to the phenotypes in this case, and a matrix of mixture coefficient C with the coefficients (or weights) of the subgraphs in each phenotype (Figure 1). The factorization was done by iteratively updating F and C using the SNMF/L algorithm (see below) to gradually reduce the error between M and $F \times C$. Once the NMF was completed, the final phenotypes were clinically characterized given the highly interpretable nature of the algorithm. The following subsections explain these procedures in more detail.

Subgraph Mining. Node-edge list graphs were prepared for patients with MODS by mapping each pSOFA subscore at days 1, 2, and 3 as nodes, and the edges as the transitions between days 1 to 2 and 2 to 3. Edges were labeled with the direction of change between subsequent nodes, and were not day-sensitive. Common subgraphs were extracted from the node-edge list graphs generated using the Molecular Substructure Miner (MoSS).[4] The MoSS search algorithm was run with no complement group and with no frequency requirement to mine as many potential subgraphs as possible. The number of subgraphs was then reduced by eliminating: (1) small subgraphs contained within larger subgraphs in order to reduce double-counting; (2) subgraphs that were static and did not have a maximum subscore of more than 2 in order to reduce the influence of organs with mild or no dysfunction and no change; and (3) subgraphs present in less than 20 patients in order to focus on more representative subgraphs. Patients who had no subgraphs remaining after the above step were eliminated from the remainder of the analysis.

Non-negative Matrix Factorization. The resulting matrix of patient-subgraph counts was then partitioned into derivation and validation splits at an 80/20 ratio. Non-negative matrix factorization (NMF) was then implemented on the derivation set enforcing sparsity using the SNMF/L algorithm with non-negative double singular value decomposition seeding previously described.[2,4] The total number of phenotypes was determined using a combination of cophenetic correlation, Akaike Information Criterion (AIC), and a pragmatic approach based on clinical interpretability and phenotype group size. This resulted in a matrix of hidden features (F) and mixture coefficient matrix (C) as illustrated in Figure 1.

Phenotype Characterization. The most representative subgraphs for each phenotype were chosen from the mixture coefficient matrix of the NMF model such that the selected subgraphs accounted for at least >75% of the phenotype group coefficients. These representative subgraphs were then used to characterize the phenotypes based on the most distinctive characteristics using the organ dysfunction type, severity, and trajectory in the first 3 days of PICU stay.

Validation. Patients in the derivation and validation set were assigned phenotype membership based on the highest probability group in the mixture coefficient matrix from the initial NMF model using the derivation set. Patients assigned to each phenotype were then compared across the derivation and validation sets based on the distribution of the maximum pSOFA subscores per day, the distribution of common laboratory test results, and the outcomes associated with each phenotype. Phenotypes with distinct characteristics and associated outcomes that

© 2020 Pinto-Sanchez LN et al. *JAMA Network Open.*

were reproducible across the derivation and validation sets were considered valid. Patients from the derivation and validation sets that belonged to the same MODS phenotype were grouped together for the remainder of the analyses.

As a sensitivity analysis to determine external validity of the phenotypes, the same procedures were conducted but using patients with MODS in PICU A as the derivation set to train a new mixture coefficient matrix and patients with MODS in PICU B as the external validation set. PICU A was the study site with the highest absolute number of MODS patients and PICU B was the site with absolute lowest number of MODS patients. The comparisons between patients in the different phenotypes in the sensitivity analysis were performed in the same way as in the main analysis.

Expanded methods for the analysis of therapeutic relevance of the MODS phenotypes in patients with vasoactive-dependent shock

Vasoactive-dependent shock was defined as the use of dopamine >5 mcg/kg/min or any dose of epinephrine or norepinephrine infusions in patients with MODS. The treatment of interest was intravenous (IV) hydrocortisone. Treatment was considered receiving 2mg/kg/day or more of IV hydrocortisone starting with 24 hour of vasoactive infusion initiation.

To balance the confounders across treatment groups, we first performed a propensity score matching of patients based on the propensity to receive IV hydrocortisone. The package *MatchIt* was used to perform the propensity score matching in R. Patients were matched 1:1 without replacement using the nearest neighbor approach. A caliper of 0.25 standard deviations of the logit of the propensity score was used to find a common region of support for the analyses. We matched patients based on the propensity to use IV hydrocortisone based on age, immunocompromised state, PRISM-III score, study site, and phenotype membership. Any treated and untreated patients outside the common region of support were not matched.

After ensuring covariate balance in the resulting matched cohort, we performed an interaction effects analysis for in-hospital mortality and vasoactive-free days using phenotype membership, hydrocortisone treatment group, and their interaction as covariates to determine whether there was a differential treatment effect associated with phenotype membership. We used logistic regression to test the associated effect of the interaction on in-hospital mortality and Poisson regression to test the associated effect of the interaction on ventilator-free days. The interaction between IV hydrocortisone and phenotype membership was performed using the phenotype with lowest mortality as reference and the analysis of deviance to test for global significance.

© 2020 Pinto-Sanchez LN et al. *JAMA Network Open.*

eFigure 1. Alluvial Plots of Organ Dysfunction Burden by Day in the 4 MODS Phenotypes. Cumulative organ dysfunction burden in each phenotype by day ordered by the highest relative contribution from top to bottom. Abbreviations: *MODS*, multiple organ dysfunction syndrome; *pSOFA*, Pediatric Sequential Organ Failure Assessment.

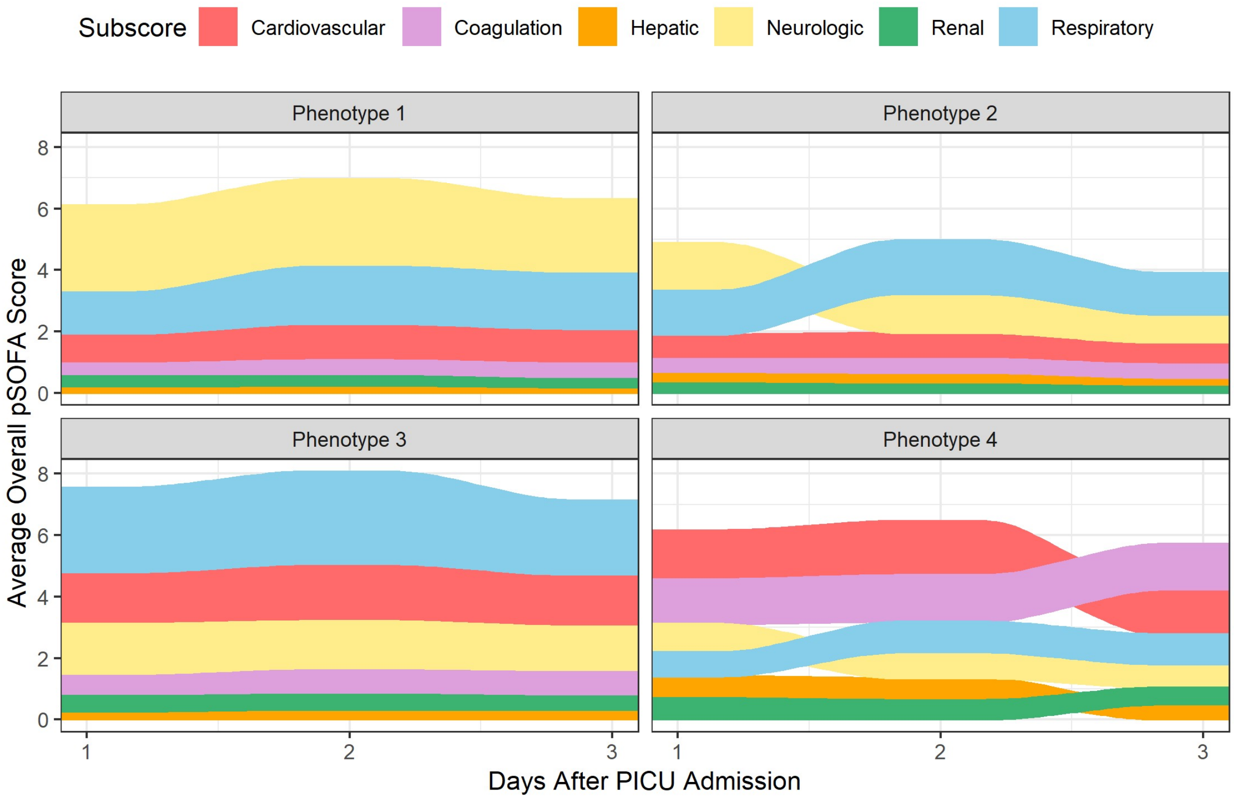

© 2020 Pinto-Sanchez LN et al. *JAMA Network Open.*

eFigure 2. Kaplan-Meier Survival Curves for the First 28 Days for the 4 MODS Phenotypes. Patients discharged alive were considered “at risk” throughout the 28-day period. The median time-to-death in non-survivors were Phenotype 1: 3.5 days (inter-quartile range 1.9, 12.4 days); Phenotype 2: 13.6 days (6.6, 34.5 days); Phenotype 3: 2 days (0.8, 9.2); Phenotype 4: 7.7 days (2.2, 22.1 days)

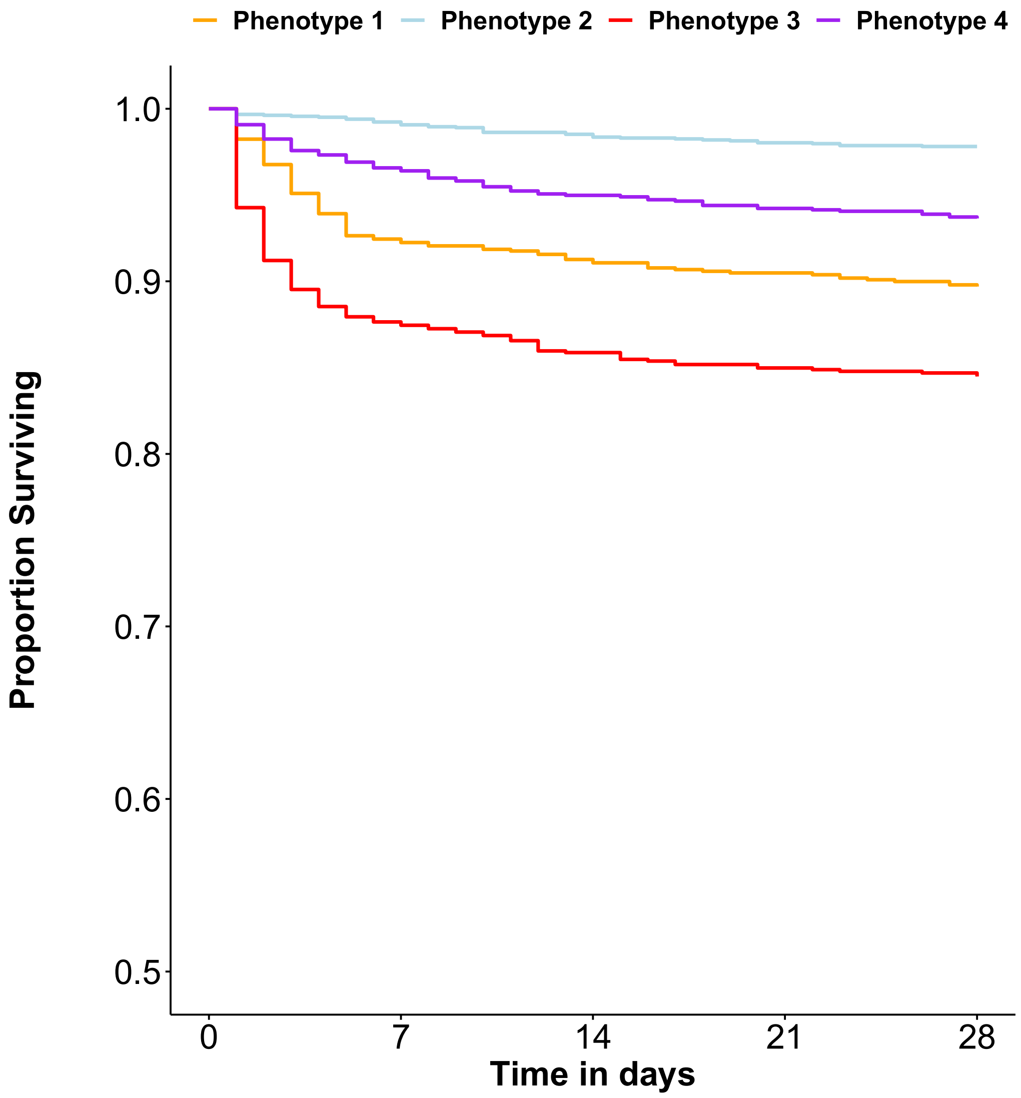

Number at risk

Time in days	0	7	14	21	28
Phenotype 1	1019	942	930	922	915
Phenotype 2	1828	1814	1801	1792	1788
Phenotype 3	1012	887	869	860	857
Phenotype 4	1195	1154	1135	1126	1120

© 2020 Pinto-Sanchez LN et al. *JAMA Network Open.*

eFigure 3. Covariate Balance in the Propensity Score Matched Cohort of Patients With Vasoactive-Dependent Shock Who Received and Did Not Receive Intravenous Hydrocortisone. The vertical dashed line represents a threshold of 0.1 absolute standardized mean difference. Abbreviations: *PRISM III,* Pediatric Risk of Mortality III score; *Immunocomp*, immunocompromised state.

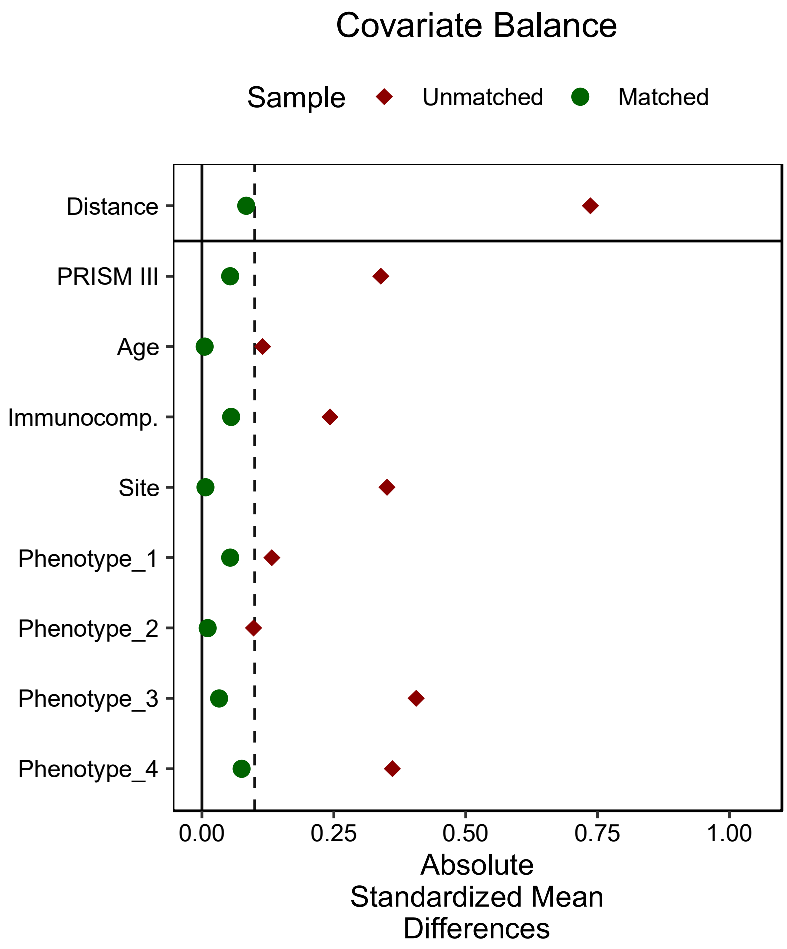

© 2020 Pinto-Sanchez LN et al. *JAMA Network Open.*

eTable 1. Maximum pSOFA Subscores Achieved by Day in Patients Within Each of the 4 MODS Phenotypes in the Derivation and Validation Sets

pSOFA Subscore, No. (%)	Score	Phenotype 1		Phenotype 2		Phenotype 3		Phenotype 4	
		Derivation (n=802)	Validation (n=217)	Derivation (n=1463)	Validation (n=365)	Derivation (n=823)	Validation (n=189)	Derivation (n=956)	Validation (n=239)
Respiratory - Day 1	0	249 (31.0)	77 (35.5)	448 (30.6)	125 (34.2)	130 (15.8)	26 (13.8)	640 (66.9)	152 (63.6)
	1	68 (8.5)	16 (7.4)	43 (2.9)	9 (2.5)	14 (1.7)	2 (1.1)	33 (3.5)	7 (2.9)
	2	230 (28.7)	57 (26.3)	279 (19.1)	65 (17.8)	37 (4.5)	6 (3.2)	105 (11.0)	31 (13.0)
	3	155 (19.3)	38 (17.5)	686 (46.9)	164 (44.9)	2 (0.2)	0 (0.0)	67 (7.0)	20 (8.4)
	4	100 (12.5)	29 (13.4)	7 (0.5)	2 (0.5)	640 (77.8)	155 (82.0)	111 (11.6)	29 (12.1)
Respiratory - Day 2	0	129 (16.1)	45 (20.7)	377 (25.8)	100 (27.4)	116 (14.1)	23 (12.2)	575 (60.1)	127 (53.1)
	1	51 (6.4)	8 (3.7)	35 (2.4)	10 (2.7)	9 (1.1)	0 (0.0)	35 (3.7)	13 (5.4)
	2	270 (33.7)	75 (34.6)	263 (18.0)	66 (18.1)	20 (2.4)	5 (2.6)	121 (12.7)	38 (15.9)
	3	163 (20.3)	34 (15.7)	757 (51.7)	184 (50.4)	4 (0.5)	0 (0.0)	78 (8.2)	26 (10.9)
	4	189 (23.6)	55 (25.3)	31 (2.1)	5 (1.4)	674 (81.9)	161 (85.2)	147 (15.4)	35 (14.6)
Respiratory - Day 3	0	176 (21.9)	61 (28.1)	455 (31.1)	106 (29.0)	145 (17.6)	31 (16.4)	561 (58.7)	120 (50.2)
	1	56 (7.0)	7 (3.2)	75 (5.1)	22 (6.0)	16 (1.9)	2 (1.1)	59 (6.2)	20 (8.4)
	2	249 (31.0)	66 (30.4)	402 (27.5)	113 (31.0)	88 (10.7)	22 (11.6)	116 (12.1)	42 (17.6)
	3	125 (15.6)	25 (11.5)	454 (31.0)	107 (29.3)	243 (29.5)	48 (25.4)	104 (10.9)	28 (11.7)
	4	196 (24.4)	58 (26.7)	77 (5.3)	17 (4.7)	331 (40.2)	86 (45.5)	116 (12.1)	29 (12.1)
Cardiovascular - Day 1	0	153 (19.1)	43 (19.8)	327 (22.4)	93 (25.5)	93 (11.3)	21 (11.1)	150 (15.7)	45 (18.8)
	1	515 (64.2)	139 (64.1)	984 (67.3)	235 (64.4)	420 (51.0)	99 (52.4)	312 (32.6)	76 (31.8)
	2	34 (4.2)	6 (2.8)	92 (6.3)	20 (5.5)	28 (3.4)	10 (5.3)	39 (4.1)	15 (6.3)
	3	24 (3.0)	15 (6.9)	21 (1.4)	7 (1.9)	21 (2.6)	7 (3.7)	453 (47.4)	102 (42.7)
	4	76 (9.5)	14 (6.5)	39 (2.7)	10 (2.7)	261 (31.7)	52 (27.5)	2 (0.2)	1 (0.4)
Cardiovascular - Day 2	0	127 (15.8)	32 (14.7)	258 (17.6)	77 (21.1)	75 (9.1)	13 (6.9)	142 (14.9)	41 (17.2)
	1	511 (63.7)	139 (64.1)	1037 (70.9)	246 (67.4)	402 (48.8)	94 (49.7)	307 (32.1)	73 (30.5)
	2	40 (5.0)	8 (3.7)	97 (6.6)	21 (5.8)	31 (3.8)	11 (5.8)	42 (4.4)	16 (6.7)
	3	27 (3.4)	17 (7.8)	29 (2.0)	8 (2.2)	40 (4.9)	14 (7.4)	457 (47.8)	108 (45.2)
	4	97 (12.1)	21 (9.7)	42 (2.9)	13 (3.6)	275 (33.4)	57 (30.2)	8 (0.8)	1 (0.4)
Cardiovascular - Day 3	0	167 (20.8)	41 (18.9)	476 (32.5)	125 (34.2)	121 (14.7)	23 (12.2)	159 (16.6)	43 (18.0)
	1	458 (57.1)	132 (60.8)	827 (56.5)	203 (55.6)	346 (42.0)	82 (43.4)	296 (31.0)	72 (30.1)
	2	43 (5.4)	9 (4.1)	91 (6.2)	22 (6.0)	42 (5.1)	11 (5.8)	95 (9.9)	23 (9.6)
	3	58 (7.2)	13 (6.0)	41 (2.8)	6 (1.6)	145 (17.6)	37 (19.6)	393 (41.1)	98 (41.0)
	4	76 (9.5)	22 (10.1)	28 (1.9)	9 (2.5)	169 (20.5)	36 (19.0)	13 (1.4)	3 (1.3)
Coagulation - Day 1	0	602 (75.1)	157 (72.4)	1059 (72.4)	251 (68.8)	498 (60.5)	115 (60.8)	366 (38.3)	89 (37.2)
	1	74 (9.2)	25 (11.5)	132 (9.0)	41 (11.2)	115 (14.0)	25 (13.2)	135 (14.1)	25 (10.5)
	2	73 (9.1)	21 (9.7)	184 (12.6)	42 (11.5)	96 (11.7)	22 (11.6)	147 (15.4)	42 (17.6)

© 2020 Pinto-Sanchez LN et al. *JAMA Network Open.*

	3	36 (4.5)	8 (3.7)	68 (4.6)	17 (4.7)	71 (8.6)	10 (5.3)	168 (17.6)	44 (18.4)
	4	17 (2.1)	6 (2.8)	20 (1.4)	14 (3.8)	43 (5.2)	17 (9.0)	140 (14.6)	39 (16.3)
Coagulation - Day 2	0	559 (69.7)	147 (67.7)	1007 (68.8)	246 (67.4)	468 (56.9)	104 (55.0)	325 (34.0)	81 (33.9)
	1	90 (11.2)	24 (11.1)	150 (10.3)	36 (9.9)	98 (11.9)	28 (14.8)	139 (14.5)	26 (10.9)
	2	97 (12.1)	30 (13.8)	213 (14.6)	51 (14.0)	128 (15.6)	26 (13.8)	157 (16.4)	45 (18.8)
	3	37 (4.6)	10 (4.6)	72 (4.9)	21 (5.8)	81 (9.8)	13 (6.9)	192 (20.1)	47 (19.7)
	4	19 (2.4)	6 (2.8)	21 (1.4)	11 (3.0)	48 (5.8)	18 (9.5)	143 (15.0)	40 (16.7)
Coagulation - Day 3	0	560 (69.8)	148 (68.2)	1026 (70.1)	254 (69.6)	479 (58.2)	106 (56.1)	327 (34.2)	86 (36.0)
	1	90 (11.2)	25 (11.5)	156 (10.7)	35 (9.6)	92 (11.2)	25 (13.2)	143 (15.0)	28 (11.7)
	2	93 (11.6)	28 (12.9)	199 (13.6)	48 (13.2)	120 (14.6)	25 (13.2)	179 (18.7)	51 (21.3)
	3	41 (5.1)	12 (5.5)	63 (4.3)	23 (6.3)	92 (11.2)	18 (9.5)	198 (20.7)	42 (17.6)
	4	18 (2.2)	4 (1.8)	19 (1.3)	5 (1.4)	40 (4.9)	15 (7.9)	109 (11.4)	32 (13.4)
Hepatic – Day 1	0	704 (87.8)	193 (88.9)	1226 (83.8)	316 (86.6)	683 (83.0)	156 (82.5)	616 (64.4)	159 (66.5)
	1	29 (3.6)	11 (5.1)	49 (3.3)	8 (2.2)	49 (6.0)	12 (6.3)	101 (10.6)	21 (8.8)
	2	48 (6.0)	10 (4.6)	135 (9.2)	23 (6.3)	68 (8.3)	15 (7.9)	136 (14.2)	24 (10.0)
	3	9 (1.1)	2 (0.9)	27 (1.8)	9 (2.5)	17 (2.1)	5 (2.6)	55 (5.8)	18 (7.5)
	4	12 (1.5)	1 (0.5)	26 (1.8)	9 (2.5)	6 (0.7)	1 (0.5)	48 (5.0)	17 (7.1)
Hepatic – Day 2	0	690 (86.0)	188 (86.6)	1217 (83.2)	314 (86.0)	663 (80.6)	152 (80.4)	602 (63.0)	151 (63.2)
	1	35 (4.4)	12 (5.5)	53 (3.6)	10 (2.7)	54 (6.6)	10 (5.3)	104 (10.9)	22 (9.2)
	2	54 (6.7)	14 (6.5)	141 (9.6)	22 (6.0)	76 (9.2)	19 (10.1)	145 (15.2)	32 (13.4)
	3	13 (1.6)	2 (0.9)	32 (2.2)	10 (2.7)	27 (3.3)	7 (3.7)	58 (6.1)	17 (7.1)
	4	10 (1.2)	1 (0.5)	20 (1.4)	9 (2.5)	3 (0.4)	1 (0.5)	47 (4.9)	17 (7.1)
Hepatic – Day 3	0	714 (89.0)	191 (88.0)	1263 (86.3)	323 (88.5)	670 (81.4)	157 (83.1)	715 (74.8)	178 (74.5)
	1	24 (3.0)	11 (5.1)	48 (3.3)	8 (2.2)	50 (6.1)	6 (3.2)	65 (6.8)	13 (5.4)
	2	41 (5.1)	12 (5.5)	100 (6.8)	20 (5.5)	72 (8.7)	14 (7.4)	88 (9.2)	18 (7.5)
	3	16 (2.0)	2 (0.9)	35 (2.4)	7 (1.9)	26 (3.2)	10 (5.3)	50 (5.2)	14 (5.9)
	4	7 (0.9)	1 (0.5)	17 (1.2)	7 (1.9)	5 (0.6)	2 (1.1)	38 (4.0)	16 (6.7)
Neurologic - Day 1	0	94 (11.7)	24 (11.1)	432 (29.5)	91 (24.9)	307 (37.3)	69 (36.5)	544 (56.9)	137 (57.3)
	1	58 (7.2)	23 (10.6)	348 (23.8)	95 (26.0)	137 (16.6)	33 (17.5)	155 (16.2)	36 (15.1)
	2	27 (3.4)	8 (3.7)	115 (7.9)	32 (8.8)	37 (4.5)	5 (2.6)	72 (7.5)	12 (5.0)
	3	8 (1.0)	4 (1.8)	342 (23.4)	87 (23.8)	106 (12.9)	20 (10.6)	44 (4.6)	22 (9.2)
	4	615 (76.7)	158 (72.8)	226 (15.4)	60 (16.4)	236 (28.7)	62 (32.8)	141 (14.7)	32 (13.4)
Neurologic - Day 2	0	86 (10.7)	24 (11.1)	357 (24.4)	74 (20.3)	275 (33.4)	62 (32.8)	481 (50.3)	119 (49.8)
	1	40 (5.0)	13 (6.0)	419 (28.6)	116 (31.8)	160 (19.4)	35 (18.5)	197 (20.6)	56 (23.4)
	2	22 (2.7)	7 (3.2)	120 (8.2)	34 (9.3)	38 (4.6)	6 (3.2)	80 (8.4)	14 (5.9)
	3	3 (0.4)	1 (0.5)	351 (24.0)	86 (23.6)	94 (11.4)	16 (8.5)	45 (4.7)	17 (7.1)
	4	651 (81.2)	172 (79.3)	216 (14.8)	55 (15.1)	256 (31.1)	70 (37.0)	153 (16.0)	33 (13.8)
Neurologic - Day 3	0	153 (19.1)	47 (21.7)	675 (46.1)	160 (43.8)	324 (39.4)	80 (42.3)	498 (52.1)	129 (54.0)

© 2020 Pinto-Sanchez LN et al. *JAMA Network Open.*

	1	79 (9.9)	18 (8.3)	343 (23.4)	98 (26.8)	167 (20.3)	32 (16.9)	262 (27.4)	71 (29.7)
	2	50 (6.2)	11 (5.1)	125 (8.5)	30 (8.2)	45 (5.5)	10 (5.3)	33 (3.5)	4 (1.7)
	3	44 (5.5)	11 (5.1)	192 (13.1)	50 (13.7)	72 (8.7)	18 (9.5)	41 (4.3)	9 (3.8)
	4	476 (59.4)	130 (59.9)	128 (8.7)	27 (7.4)	215 (26.1)	49 (25.9)	122 (12.8)	26 (10.9)
Renal – Day 1	0	612 (76.3)	175 (80.6)	1188 (81.2)	294 (80.5)	577 (70.1)	133 (70.4)	673 (70.4)	173 (72.4)
	1	123 (15.3)	19 (8.8)	162 (11.1)	42 (11.5)	114 (13.9)	31 (16.4)	99 (10.4)	29 (12.1)
	2	37 (4.6)	13 (6.0)	58 (4.0)	15 (4.1)	72 (8.7)	13 (6.9)	52 (5.4)	10 (4.2)
	3	15 (1.9)	5 (2.3)	17 (1.2)	7 (1.9)	33 (4.0)	11 (5.8)	16 (1.7)	3 (1.3)
	4	15 (1.9)	5 (2.3)	38 (2.6)	7 (1.9)	27 (3.3)	1 (0.5)	116 (12.1)	24 (10.0)
Renal – Day 2	0	611 (76.2)	174 (80.2)	1169 (79.9)	292 (80.0)	550 (66.8)	126 (66.7)	668 (69.9)	173 (72.4)
	1	117 (14.6)	21 (9.7)	177 (12.1)	44 (12.1)	138 (16.8)	37 (19.6)	95 (9.9)	26 (10.9)
	2	41 (5.1)	11 (5.1)	58 (4.0)	16 (4.4)	69 (8.4)	10 (5.3)	60 (6.3)	12 (5.0)
	3	15 (1.9)	5 (2.3)	21 (1.4)	6 (1.6)	38 (4.6)	11 (5.8)	14 (1.5)	5 (2.1)
	4	18 (2.2)	6 (2.8)	38 (2.6)	7 (1.9)	28 (3.4)	5 (2.6)	119 (12.4)	23 (9.6)
Renal – Day 3	0	652 (81.3)	181 (83.4)	1253 (85.6)	317 (86.8)	602 (73.1)	136 (72.0)	706 (73.8)	185 (77.4)
	1	91 (11.3)	23 (10.6)	120 (8.2)	32 (8.8)	119 (14.5)	32 (16.9)	85 (8.9)	26 (10.9)
	2	31 (3.9)	5 (2.3)	46 (3.1)	8 (2.2)	45 (5.5)	11 (5.8)	57 (6.0)	8 (3.3)
	3	11 (1.4)	2 (0.9)	19 (1.3)	4 (1.1)	30 (3.6)	4 (2.1)	26 (2.7)	3 (1.3)
	4	17 (2.1)	6 (2.8)	25 (1.7)	4 (1.1)	27 (3.3)	6 (3.2)	82 (8.6)	17 (7.1)

Abbreviations: *MODS*, multiple organ dysfunction syndrome; *pSOFA*, Pediatric Sequential Organ Failure Assessment.

© 2020 Pinto-Sanchez LN et al. *JAMA Network Open.*

eTable 2. Laboratory Test Results by Day 3 of PICU Stay in the 4 MODS Phenotypes Across the Derivation and Validation Sets

Laboratory Test Results by Day 3, Median (IQR)	Missing values	Phenotype 1		Phenotype 2		Phenotype 3		Phenotype 4	
		Derivation (n=802)	Validation (n=217)	Derivation (n=1,463)	Validation (n=365)	Derivation (n=823)	Validation (n=189)	Derivation (n=956)	Validation (n=239)
Min. White Blood Cell Count, K/uL	7.9%	7.9 (5.3, 11)	7.9 (5, 11.1)	8.2 (5.5, 12)	8.6 (5.8, 12)	7 (3.8, 11)	6.9 (3.9, 9.9)	6 (2.6, 9.4)	5.7 (2.1, 9.5)
Min. Lymphocyte Count, K/uL	10.6%	1.3 (0.7, 2.2)	1.2 (0.8, 2.2)	1.3 (0.7, 2.2)	1.4 (0.7, 2.4)	1.1 (0.5, 1.9)	1.2 (0.5, 2.1)	0.8 (0.3, 1.5)	0.8 (0.2, 1.5)
Max. Blood Urea Nitrogen, mg/dL	4.5%	12 (8, 18)	12 (8, 17)	12 (8, 18)	12 (8, 17)	14 (9, 23)	13 (9, 22)	15 (10, 29)	16 (10, 28)
Max. Creatinine, mg/dL	4.4%	0.4 (0.3, 0.6)	0.4 (0.3, 0.6)	0.4 (0.2, 0.6)	0.4 (0.3, 0.6)	0.4 (0.3, 0.8)	0.4 (0.3, 0.8)	0.5 (0.3, 0.9)	0.5 (0.3, 0.9)
Min. Albumin, g/dL	29.8%	3.1 (2.5, 3.6)	3.1 (2.5, 3.6)	3.1 (2.6, 3.7)	3.2 (2.7, 3.8)	2.7 (2.2, 3.3)	2.6 (2.2, 3.2)	2.8 (2.3, 3.4)	2.8 (2.2, 3.4)
Max. Aspartate Aminotransferase, U/L	38.8%	53 (33, 136)	54 (29, 176)	51 (30, 111)	42 (28, 100)	59 (32, 210)	72 (36, 184)	62 (32, 142)	61 (33, 129)
Max. Alanine Aminotransferase, U/L	38.8%	27 (18, 80)	33 (18, 82)	28 (17, 74)	25 (16, 59)	35 (18, 123)	45 (21, 121)	37 (18, 98)	38 (19, 93)
Max. Total Bilirubin, mg/dL	37.9%	0.5 (0.3, 1.2)	0.4 (0.2, 1)	0.6 (0.2, 2.3)	0.4 (0.3, 1.4)	0.6 (0.2, 1.5)	0.6 (0.3, 1.7)	1.2 (0.5, 3)	1.2 (0.5, 4.1)
Min. Hemoglobin, g/dL	6.4%	9.2 (7.5, 11)	8.9 (7.2, 11)	9.5 (7.8, 11)	9.9 (7.7, 12)	8.8 (7.2, 11)	8.8 (7.2, 11)	8.3 (6.9, 10)	8.3 (7.1, 10)
Max. Platelet Count, K/uL	8.1%	186 (107, 267)	178 (101, 272)	188 (95, 272)	188 (91, 267)	155 (68, 244)	158 (68, 269)	84 (28, 181)	78 (24, 176)
Max. International Normalized ratio	40.3%	1.4 (1.2, 1.7)	1.4 (1.2, 1.8)	1.3 (1.2, 1.6)	1.3 (1.2, 1.5)	1.5 (1.2, 2.1)	1.4 (1.2, 2)	1.4 (1.2, 1.7)	1.4 (1.2, 1.9)
Max. Partial Thromboplastin Time	40.5%	35 (30, 45)	35 (30, 44.5)	35 (30, 45)	33 (30, 41)	40 (31, 59)	41 (33, 66)	38 (31, 53)	39 (32, 56)
Max. Lactate, mmol/L	36.1%	2 (1.2, 3.7)	2.4 (1.2, 5.1)	1.6 (1.1, 2.9)	1.7 (1.1, 2.8)	2.5 (1.4, 5.7)	2.6 (1.4, 6)	2 (1.3, 3.8)	2 (1.2, 3.8)

Abbreviations: *IQR*, inter-quartile range.

© 2020 Pinto-Sanchez LN et al. *JAMA Network Open.*

eTable 3. Outcomes Associated With the 4 MODS Phenotypes Across the Derivation and Validation Sets

Outcomes	Phenotype 1		Phenotype 2		Phenotype 3		Phenotype 4	
	**Derivation (n=802)**	**Validation (n=217)**	**Derivation (n=1463)**	**Validation (n=365)**	**Derivation (n=823)**	**Validation (n=189)**	**Derivation (n=956)**	**Validation (n=239)**
Length of Stay, Days (IQR)[a]	10 (4.7, 20.9)	9 (4.3, 19.5)	8.2 (4.6, 15.6)	7.8 (4, 14.2)	11.3 (4.9, 22.4)	11.1 (4.7, 21.9)	10 (5.8, 18.6)	9.9 (5.5, 19.9)
MODS on Day 7, No. (%)	269 (33.5)	85 (39.2)	233 (15.9)	58 (15.9)	336 (40.8)	81 (42.9)	255 (26.7)	82 (34.3)
In-Hospital Mortality, No. (%)	88 (11.0)	28 (12.9)	47 (3.2)	13 (3.6)	145 (17.6)	31 (16.4)	69 (7.2)	25 (10.5)

[a]Presented in medians and inter-quartile ranges (IQR).
Abbreviations: *MODS*, multiple organ dysfunction syndrome.

© 2020 Pinto-Sanchez LN et al. *JAMA Network Open.*

eTable 4. Laboratory Test Results and Outcomes Associated With the MODS Phenotypes When Using Patients in Hospital A as the Derivation Set and Patients in Hospital B as an External Validation Set

	Phenotype 1		Phenotype 2		Phenotype 3		Phenotype 4	
	**Derivation (n=471)**	**Validation (n=548)**	**Derivation (n=1,053)**	**Validation (n=775)**	**Derivation (n=643)**	**Validation (n=369)**	**Derivation (n=846)**	**Validation (n=349)**
**Max. pSOFA Subscores by Day 3[a]**								
Respiratory	3 (2, 4)	3 (3, 4)	3 (2, 3)	3 (2, 4)	4 (4, 4)	4 (4, 4)	0 (0, 3)	2 (0, 4)
Cardiovascular	1 (1, 1)	1 (1, 1)	1 (1, 1)	1 (1, 1)	1 (1, 3)	1 (1, 4)	2 (1, 3)	2 (1, 3)
Coagulation	0 (0, 1)	0 (0, 1)	0 (0, 2)	0 (0, 1)	0 (0, 2)	1 (0, 2)	2 (0, 3)	2 (0, 3)
Hepatic	0 (0, 0)	0 (0, 0)	0 (0, 0)	0 (0, 0)	0 (0, 0)	0 (0, 0)	0 (0, 2)	0 (0, 2)
Neurologic	3 (2, 3)	4 (4, 4)	3 (2, 3)	3 (3, 4)	3 (2, 3)	4 (3, 4)	2 (2, 3)	3 (1, 4)
Renal	0 (0, 0)	0 (0, 1)	0 (0, 0)	0 (0, 1)	0 (0, 0)	1 (0, 2)	0 (0, 1)	0 (0, 2)
**Laboratory Test Results by Day 3[a]**								
Min. White Blood Cell Count, K/uL	7.9 (5.2, 10.9)	8 (5.3, 11)	7.9 (5.3, 11.3)	8.8 (5.9, 11.8)	6.8 (3.8, 10.6)	7.2 (3.8, 11.1)	5.4 (2.1, 8.9)	7 (3.8, 10.9)
Min. Lymphocyte Count, K/uL	1.3 (0.7, 2.3)	1.3 (0.8, 2.2)	1.2 (0.6, 2.2)	1.4 (0.8, 2.3)	1 (0.5, 1.9)	1.2 (0.5, 2.1)	0.7 (0.3, 1.3)	1.1 (0.5, 1.9)
Max. Blood Urea Nitrogen, mg/dL	11 (7, 16)	12 (9, 19)	12 (8, 19)	12 (8, 17)	13 (8, 23.3)	14 (9, 22)	16 (10, 29.8)	15 (10, 27)
Max. Creatinine, mg/dL	0.3 (0.2, 0.5)	0.4 (0.3, 0.7)	0.3 (0.2, 0.6)	0.4 (0.3, 0.6)	0.4 (0.3, 0.7)	0.5 (0.3, 0.8)	0.5 (0.3, 0.9)	0.5 (0.4, 0.9)
Min. Albumin, g/dL	3 (2.3, 3.5)	3.1 (2.6, 3.7)	2.9 (2.4, 3.4)	3.4 (2.9, 3.9)	2.5 (2, 3.2)	2.9 (2.4, 3.5)	2.7 (2.2, 3.2)	3.1 (2.6, 3.8)
Max. Aspartate Aminotransferase,U/L	52 (31, 147)	54 (32, 147)	58 (33, 153)	43 (28, 83)	58 (31, 211)	68 (38, 190)	62 (31, 151)	63 (35, 131)
Max. Alanine Aminotransferase,U/L	36 (20, 112)	25 (17, 63)	37 (19, 109)	22 (15, 50)	36 (19, 126)	37 (19, 108)	44 (20, 115)	27 (16, 70)
Max. Total Bilirubin, mg/dL	0.5 (0.3, 1)	0.5 (0.3, 1.3)	0.7 (0.3, 2.6)	0.4 (0.2, 1.4)	0.7 (0.3, 1.7)	0.4 (0.2, 1.3)	1.3 (0.6, 3.5)	1 (0.4, 2.7)
Min. Hemoglobin, g/dL	10.1 (8, 12)	8.4 (7.2, 9.9)	9.7 (7.7, 11.5)	9.6 (7.8, 11.1)	9 (7.5, 10.7)	8.1 (6.8, 10)	8.4 (7.1, 10.2)	8.1 (6.6, 9.8)
Max. Platelet Count, K/uL	181 (99, 272)	186 (109, 265)	176 (85, 270)	204 (115, 269)	160 (65, 258)	153 (73, 233)	76 (25, 172)	98 (36, 195)
Max. International Normalized ratio	1.3 (1.1, 1.5)	1.4 (1.2, 1.8)	1.3 (1.1, 1.6)	1.3 (1.2, 1.6)	1.4 (1.2, 1.9)	1.6 (1.3, 2.2)	1.3 (1.1, 1.)	1.5 (1.3, 1.8)
Max. Partial Thromboplastin Time	33.2 (27.5, 48.6)	35.6 (31.2, 44.1)	35.5 (28.5, 53.2)	34.2 (30.4, 40.7)	38.4 (30.1, 59.2)	42.4 (34.2, 59.4)	37.9 (29.9, 56.1)	40.4 (34.2, 51.2)
Max. Lactate, mmol/L	2.3 (1.2, 4.1)	2 (1.2, 3.8)	1.7 (1, 2.8)	1.6 (1.1, 2.9)	2.2 (1.2, 5.2)	3 (1.7, 6.3)	1.9 (1.2, 3.6)	2.3 (1.4, 4.2)
**Outcomes**								
Length of Stay, Days[a]	9.8 (4.9, 20.9)	9.8 (4.4, 20.2)	9.3 (5, 17)	6.8 (3.8, 12.6)	11.9 (5.1, 22.9)	10 (3.9, 20.7)	10 (5.8, 18.3)	10 (5.5, 19.5)
MODS on Day 7, No. (%)	118 (25.1)	236 (43.1)	132 (12.5)	159 (20.5)	216 (33.6)	201 (54.5)	205 (24.2)	132 (37.8)
In-Hospital Mortality, No. (%)	51 (10.8)	65 (11.9)	41 (3.9)	19 (2.5)	105 (16.3)	71 (19.2)	57 (6.7)	37 (10.6)

[a]Presented in medians and inter-quartile ranges (IQR).
Abbreviations: *MODS*, multiple organ dysfunction syndrome.

© 2020 Pinto-Sanchez LN et al. *JAMA Network Open.*

eTable 5. Distribution and Outcomes Associated With the 4 MODS Phenotypes Across Age Groups

Age Groups	Total No. (% within age group)	In-hospital mortality, No. (%)	Persistent MODS on day 7, No. (%)	Hospital-free days at 28 days (IQR)	Ventilator-free days at 28 days (IQR)	Vasoactive-free days at 28 days (IQR)	Median time to death in non-survivors (IQR)
**All ages**							
Phenotype 1	1019 (20.2)	116 (11.4)	320 (31.4)	16 (1, 23)	23 (13, 26)	28 (27, 28)	3.5 (1.9, 12.4)
Phenotype 2	1828 (36.2)	60 (3.3)	319 (17.5)	20 (12, 24)	26 921, 27)	28 (28, 28)	13.6 (6.6, 34.4)
Phenotype 3	1012 (20)	176 (17.4)	448 (44.3)	12 (1, 21)	20 (6, 260	28 (24, 28)	2 (0.8, 9.2)
Phenotype 4	1195 (23.6)	94 (7.9)	307 (25.7)	17 (4, 22)	27 (22, 28)	27 (26, 28)	7.7 (2.2 (22.1)
**<1 mo.**							
Phenotype 1	28 (14.2)	8 (28.6)	15 (53.6)	1 (1, 9)	18 (5, 24)	26 (0, 28)	3.4 (2.6, 8.9)
Phenotype 2	68 (34.5)	6 (8.8)	14 (20.6)	16 (8, 22)	26 (22, 28)	28 (25, 28)	20.8 (11.8, 40)
Phenotype 3	44 (22.3)	10 (22.7)	24 (54.5)	8 (1, 16)	19 (9, 23)	26 (14, 28)	4.2 (1.8, 12)
Phenotype 4	57 (28.9)	1 (1.8)	15 (26.3)	14 (1, 21)	24 (20, 28)	26 (23, 28)	0.2 (NA)
**1 to 11 mo.**							
Phenotype 1	228 (21.7)	30 (13.2)	84 (36.8)	13 (1, 20)	21 (13, 25)	28 (26, 28)	4.3 (2.7, 12.1)
Phenotype 2	411 (39.2)	10 (2.4)	86 (20.9)	18 (5, 23)	24 (19, 27)	28 (28, 28)	26 (13.1, 45.7)
Phenotype 3	238 (22.7)	35 (14.7)	110 (46.2)	10 (1, 19)	18 (2, 24)	28 (26, 28)	1.5 (0.9, 9.7)
Phenotype 4	172 (16.4)	14 (8.1)	53 (30.8)	15 (1, 21)	28 (25, 28)	28 (25, 28)	5.7 (0.8, 17.3)
**12 to 23 mo.**							
Phenotype 1	111 (20.2)	9 (8.1)	36 (32.4)	17 (2, 24)	22 (13, 26)	28 (28, 28)	3 (2, 5)
Phenotype 2	220 (40)	6 (2.7)	31 (14.1)	21 (15, 24)	26 (21, 27)	28 (28, 28)	21.5 (8.4, 43.9)
Phenotype 3	132 (24)	19 (14.4)	59 (44.7)	12 (1, 20)	19 (3, 26)	28 (25, 28)	1.7 (1, 3.3)
Phenotype 4	87 (15.8)	12 (13.8)	36 (41.4)	13 (1, 20)	27 (19, 28)	27 (25, 28)	4.2 (1.4, 10.5)
**24 to 59 mo.**							
Phenotype 1	180 (22.2)	20 (11.1)	55 (30.6)	18 (1, 23)	23 (20, 26)	28 (28, 28)	3.7 (1.8, 14.2)
Phenotype 2	291 (35.9)	6 (2.1)	40 (13.7)	21 (14, 25)	26 (23, 27)	28 (28, 28)	21.6 (14.8, 41.4)
Phenotype 3	165 (20.3)	26 (15.8)	74 (44.8)	9 91, 22)	20 (10, 25)	28 (24, 28)	2 (0.8, 5)
Phenotype 4	175 (21.6)	13 (7.4)	44 (25.1)	17 (6, 22)	27 (21, 28)	26 (26, 28)	2.4 (1.5, 7.2)
**60 to 143 mo.**							
Phenotype 1	219 (20)	27 (12.3)	62 (28.3)	18 (3, 23)	24 (15, 26)	28 (28, 28)	4.2 (1.2, 14.3)
Phenotype 2	433 (39.6)	15 (3.5)	67 (15.5)	22 (15, 24)	26 (23, 27)	28 (28, 28)	9.4 (3.6, 21.5)
Phenotype 3	200 (18.30	36 (18)	81 (40.5)	15 (1, 21)	22 (8, 26)	28 (24, 28)	1.9 (0.8, 9.2)
Phenotype 4	241 (22)	21 (8.7)	53 (22)	18 (7, 23)	27 (22, 28)	27 (26, 28)	9.3 (3, 17.1)
**144 to 215 mo.**							
Phenotype 1	219 (20)	19 (8.7)	61 (27.9)	17 (1, 22)	24 (14, 27)	28 (27, 28)	2 (0.7, 3.7)
Phenotype 2	330 (30.1)	15 (4.5)	69 (20.9)	21 (14, 24)	26 (23, 28)	28 (28, 28)	6.6 (4.9, 33.4)
Phenotype 3	187 (17.1)	41 (21.9)	83 (44.4)	15 (1, 22)	23 (10, 27)	27 (23, 28)	2.3 (0.8, 11.8)
Phenotype 4	360 (32.8)	24 (6.7)	78 (21.7)	19 (10, 22)	28 (28, 28)	27 (26, 28)	13.6 (5.3 26.8)
**≥216 mo.**							
Phenotype 1	34 (13.2)	3 (8.8)	7 (20.6)	20 (3, 24)	26 (21, 27)	28 (28, 28)	26.8 (13.4, 36.6)
Phenotype 2	75 (29.1)	2 (2.7)	12 (16)	19 (12, 24)	26 (20, 27)	28 (28, 28)	16.9 (15.5, 18.4)
Phenotype 3	46 (17.8)	9 (19.7)	17 (37)	12 (1, 22)	21 (13, 28)	27 (24, 28)	0.9 (0.5, 3.2)
Phenotype 4	103 (39.9)	9 (8.7)	28 (27.1)	17 (6, 23)	28 (26, 28)	27 (27, 28)	12.3 (10, 38.8)

Abbreviations: *IQR*, inter-quartile range; *MODS*, multiple organ dysfunction syndrome

© 2020 Pinto-Sanchez LN et al. *JAMA Network Open.*

eTable 6. Unadjusted and Adjusted Outcomes Associated With the 4 MODS Phenotypes

Outcomes	Phenotype 1: Severe, persistent encephalopathy		Phenotype 2: Moderate, resolving hypoxemia	Phenotype 3: Severe, persistent hypoxemia and shock		Phenotype 4: Moderate, persistent Thrombocytopenia and shock	
	Unadjusted	Adjusted[a]		Unadjusted	Adjusted[a]	Unadjusted	Adjusted[a]
Vasoactive-free days at 28 days, IRR (95% CI)	0.9 (0.9, 0.9)	1 (1, 1)	Reference	0.8 (0.8, 0.9)	0.9 (0.9, 1)	0.9 (0.9, 0.9)	1 (1, 1)
Ventilator-free days at 28 days, IRR (95% CI)	0.8 (0.8, 0.8)	0.9 (0.9, 0.9)	Reference	0.7 (0.7, 0.7)	0.9 (0.8, 0.9)	1 (1, 1)	1.1 (1.1, 1.1)
Hospital-free days at 28 days, IRR (95% CI)	0.8 (0.8, 0.8)	0.9 (0.9, 0.9)	Reference	0.7 (0.7, 0.7)	0.8 (0.8, 0.8)	0.8 (0.8, 0.9)	1 (1, 1)
Persistent MODS on day 7, OR (95% CI)	2.2 (1.8, 2.6)	1.6 (1.3, 1.9)	Reference	3.8 (3.2, 4.5)	2.3 (1.9, 2.8)	1.6 (1.3, 2)	1.2 (1, 1.4)
In-hospital mortality, OR (95% CI)	3.8 (2.8, 5.3)	2.3 (1.6, 3.3)	Reference	6.2 (4.6, 8.5)	2.2 (1.6, 3.2)	2.5 (1.8, 3.5)	1.3 (0.9, 1.9)

[a]Adjusted for PRISM-III score on admission, immunocompromised state, age, and study site.
Abbreviations: *PRISM-III*, Pediatric Risk of Mortality III Score; *IRR*, incidence rate ratio; *CI*, confidence interval; *OR*, odds ratios; *MODS*, multiple organ dysfunction syndrome

© 2020 Pinto-Sanchez LN et al. *JAMA Network Open.*

eTable 7. Multivariable Association Between Noncharacteristic Organ Dysfunctions and In-Hospital Mortality

Non-characteristic Organ Dysfunction[a]	Phenotype 1: Severe, persistent encephalopathy	Phenotype 2: Moderate, resolving hypoxemia	Phenotype 3: Severe, persistent hypoxemia and shock	Phenotype 4: Moderate, persistent Thrombocytopenia and shock
Respiratory, OR (95% CI)	1.5 (0.7, 3.5)	--	--	2.3 (1.4, 3.6)
Cardiovascular, OR (95% CI)	9.8 (6.3, 15.6)	3.2 (1.7, 5.7)	--	--
Coagulation, OR (95% CI)	1.9 (1.2, 3.1)	2 (1.1, 3.5)	1.9 (1.3, 2.8)	--
Hepatic, OR (95% CI)	0.9 (0.5, 1.8)	2.5 (1.4, 4.6)	1.4 (0.9, 2.2)	1.7 (1, 2.6)
Neurologic, OR (95% CI)	--	1.6 (0.7, 4.9)	2.7 (1.4, 6.1)	1.4 (0.8, 2.7)
Renal, OR (95% CI)	1.5 (0.8, 2.6)	0.9 (0.4, 2)	3.6 (2.5, 5.2)	1.4 (0.9, 2.2)

[a]Non-characteristic organ dysfunctions defined as those organ systems that were not used to characterize a specific phenotype and had a pSOFA subscore of ≥2 by day 3 of PICU stay.
Abbreviations: *MODS*, multiple organ dysfunction syndrome; *OR*, odds ratio; *CI*, confidence interval; *pSOFA*, Pediatric Sequential Organ Failure Assessment.

© 2020 Pinto-Sanchez LN et al. *JAMA Network Open.*

eReferences

1. Sanchez-Pinto LN, Luo Y, Churpek MM. Big Data and Data Science in Critical Care. *Chest*. 2018.
2. Gaujoux R, Seoighe C. A flexible R package for nonnegative matrix factorization. *BMC bioinformatics*. 2010;11(1):367.
3. Lee DD, Seung HS. Learning the parts of objects by non-negative matrix factorization. *Nature*. 1999; 401:788–791.
4. Müller F-J, Laurent LC, Kostka D, et al. Regulatory networks define phenotypic classes of human stem cell lines. *Nature*. 2008; 455:401–405.
5. Kim H, Park H. Sparse non-negative matrix factorizations via alternating non-negativity-constrained least squares for microarray data analysis. *Bioinformatics*. 2007; 23:1495–1502.
6. Gao Y, Church G. Improving molecular cancer class discovery through sparse non-negative matrix factorization. *Bioinformatics*. 2005; 21:3970–3975.
7. Luo Y, Xin Y, Joshi R, Celi LA, Szolovits P. Predicting ICU Mortality Risk by Grouping Temporal Trends from a Multivariate Panel of Physiologic Measurements. Paper presented at: *AAAI2016*.
8. Luo Y, Xin Y, Hochberg E, Joshi R, Uzuner O, Szolovits P. Subgraph augmented non-negative tensor factorization (SANTF) for modeling clinical narrative text. *Journal of the American Medical Informatics Association*. 2015 Sep 1;22(5):1009-19.
9. Liu H, Hunter L, Kešelj V, Verspoor K. Approximate subgraph matching-based literature mining for biomedical events and relations. *PloS one*. 2013;8(4).
10. Borgelt C, Meinl T, Berthold M. Moss: a program for molecular substructure mining. Paper presented at: *Proceedings of the 1st international workshop on open source data mining: frequent pattern mining implementations 2005*.

© 2020 Pinto-Sanchez LN et al. *JAMA Network Open.*
